# Effects of Resveratrol on Metabolic Indicators in Patients with Type 2 Diabetes: A Systematic Review and Meta-Analysis

**DOI:** 10.1155/2022/9734738

**Published:** 2022-01-31

**Authors:** Wei Gu, Jianlin Geng, Hang Zhao, Xiaolong Li, Guangyao Song

**Affiliations:** ^1^Graduate School of Hebei Medical University, Shijiazhuang 050017, China; ^2^Endocrinology Department, Harrison International Peace Hospital, Hengshui 053000, China; ^3^Endocrinology Department, Hebei General Hospital, Shijiazhuang 050051, China

## Abstract

**Background and Aims:**

Previous studies on the effects of resveratrol on metabolic indicators reported contradictory findings, and these indicators have not been frequently studied in patients with type 2 diabetes. In this study, we aimed to examine the effects of resveratrol on metabolic indicators in a specific group of people with type 2 diabetes using the most recent literature.

**Methods:**

We used RevMan 5.4 and Stata 14.0 software to identify randomized controlled studies on the impact of resveratrol on metabolic indicators in patients with type 2 diabetes using relevant search terms and keywords such as “resveratrol” and “type 2 diabetes” in the China National Knowledge Infrastructure, PubMed, Cochrane, and Embase. Data were expressed as the weighted mean difference (WMD) and 95% confidence interval (CI).

**Results:**

This meta-analysis included 19 studies involving 1151 patients with type 2 diabetes, including 584 patients treated with resveratrol and 567 patients who received placebo. Compared with the control data, large doses of resveratrol (≥1000 mg) reduced fasting blood glucose levels (WMD: −18.76 mg/dL, 95% CI: −23.43, −14.09; *P* < 0.00001). Additionally, resveratrol reduced systolic blood pressure (WMD: −7.97 mmHg, 95% CI: −10.63, −5.31; *P* < 0.00001) and diastolic blood pressure (WMD: −3.55 mmHg, 95% CI: −5.18, −1.93; *P* < 0.00001) in patients with type 2 diabetes but did not improve waist circumference (WMD: 0.05 cm, 95% CI: −1.77, 1.88; *P*=0.95), triglyceride levels (WMD: −4.49 mg/dL, 95% CI: −24.23, 15.25; *P*=0.66), or high-density lipoprotein cholesterol levels (WMD: −1.05 mg/dL, 95% CI: −2.44, 0.33; *P*=0.14) in patients with type 2 diabetes.

**Conclusion:**

This systematic review and meta-analysis updated the most recent literature and provided new evidence, proving that resveratrol treatment can reduce systolic blood pressure and diastolic blood pressure. High-dose resveratrol can reduce fasting blood glucose in patients with type 2 diabetes, although it has no effect on waist circumference, triglyceride, and high-density lipoprotein cholesterol.

## 1. Introduction

With recent changes in lifestyle such as diets high in sugar and fat and a lack of exercise, the prevalence of diabetes has gradually increased [1]. According to the WHO diagnostic criteria for diabetes, the latest prevalence of diabetes in China has increased to 11.2% [2]. In addition to elevated blood sugar, patients with diabetes often have manifestations of metabolic syndrome such as obesity, hypertension, and dyslipidemia [3], all of which increase the risk of diabetic complications, especially cardiovascular and cerebrovascular diseases, and ultimately lead to increased mortality [4].

At present, type 2 diabetes and its abnormal metabolism-related indicators are mainly treated using hypoglycemic, hypotensive, lipid-lowering, and insulin-sensitizing drugs, such as Metformin [5], glucagon-likepeptide-1(GLP-1) analogs [6], and sodium-glucose cotransporter-2 (SGLT-2) inhibitors [7], together with diet modification and exercise. However, Metformin [8] and GLP-1 analogs [9] cannot be tolerated by some patients because of gastrointestinal reactions. SGLT-2 inhibitor therapy is associated with risks of urinary tract infection and diabetic ketoacidosis [10].

Resveratrol is a natural polyphenolic compound. It was extracted from the roots of white melon in 1940 [11] and later detected in more than 70 plants. It has several pharmacological effects, including antioxidant, antitumor, cardioprotective, anti-inflammatory, and neuroprotective functions [12]. Some studies illustrated that resveratrol improves both insulin resistance (IR) and hepatic steatosis while lowering blood glucose [13, 14]. A meta-analysis by Haghighatdoost and Hariri [15], which covered 21 randomized controlled trials (RCTs) including 949 patients, confirmed that resveratrol reduced total cholesterol (TC) levels and increased high-density lipoprotein cholesterol (HDL-C) levels. A meta-analysis of 29 RCTs involving 1069 patients by Guo et al. [16] suggested that resveratrol can significantly reduce systolic (SBP) and diastolic blood pressure (DBP) as well as low-density lipoprotein cholesterol (LDL-C) and glycosylated hemoglobin (HbA1c) levels without significantly altering TC and triglyceride (TG) levels. Therefore, the effect of resveratrol on glucose and lipid metabolism remains controversial. In the present study, we limited our observations to a specific group of people with type 2 diabetes, using the most recent literature, and examined the effects of resveratrol on metabolic indicators such as waist circumference (WC), fasting blood glucose (FBG), blood pressure (BP), TG, and HDL-C, which are the primary diagnostic indicators of metabolic syndrome.

## 2. Methods

This systematic review and meta-analysis was performed in accordance with PRISMA (Preferred Reporting Items for Systematic Reviews and Meta-Analyses) statement guidelines [17] and the PICOS model for the definition of the inclusion criteria: P (Population): “Type 2 diabetes patients”, I (Intervention): “impact of the of resveratrol supplementation”, C (Comparators): “same conditions with control or placebo”, O (Outcome): “metabolic syndrome related indicators”, and S (study design): “randomized controlled trials”.

### 2.1. Search Strategy

Studies published through August 1, 2021, were retrieved from China National Knowledge Infrastructure, PubMed, Cochrane Library, and Embase using the following keywords: “type 2 diabetes”, “resveratrol”, “trans resveratrol”, “blood sugar”, “blood pressure”, “waist circumference”, “lipid”, “triglyceride”, “high density lipoprotein”, “type 2 diabet^*∗*^”, “resveratrol”, “trans-resveratrol”, “FBG”, “fast blood-glucose”, “glucose”, “SBP”, “DBP”, “BP”, “WC”, “waist”, “fat”, “lipid”, “triglycerides”, “HDL-C”, “blood fats”, and “lipid”. The search methods included a search of similar keywords, subject word retrieval, arbitrary word retrieval, and topic and abstract retrieval to obtain the most complete list of relevant documents. In addition, panel discussions were held to resolve any differences. Quotes, parentheses, and asterisks were used to search for exact terms, locate a set of search terms, and identify all words derived from a single keyword. No restriction on publication time and language was employed. This study was prospectively registered in the International Prospective Registry for Systematic Reviews (PROSPERO) and approved under the protocol number CRD42021286314.

### 2.2. Inclusion and Exclusion Criteria

We evaluated the effects of resveratrol supplementation compared to placebo in the treatment and prevention of type 2 diabetes. The included patients with type 2 diabetes met the diagnostic criteria for diabetes published by WHO in 1999 [18], including diabetes symptoms (typical symptoms include “three more and one less”) plus one of the following three diagnostic criteria: random blood glucose (blood glucose at any time of the day) ≥ 11.1 mmol/L (200 mg/dL); fasting blood sugar (FBG; fasting refers to a period of at least 8 h without consuming calories) ≥ 7.0 mmol/L (126 mg/dL); and 2-hour blood glucose ≥11.1 mmol/L (200 mg/dL) after glucose loading.

The study inclusion criteria were as follows: randomized, parallel, or crossover placebo-controlled clinical study (i.e., RCT); subjects diagnosed with type 2 diabetes; and complete data were available for each group after the intervention or complete relevant data could be obtained via data conversion. Meanwhile, the following studies were excluded: reviews, peer reviews, letters, conference papers, case reports, and animal and cell studies and RCTs with only abstracts and no full-text data, unreliable data, or incomplete data.

### 2.3. Data Extraction and Quality Evaluation

Data from the included trials were extracted independently by two authors (Wei Gu and Xiaolong Li) and the inconsistencies were resolved with a senior researcher (Jianlin Geng). The inclusion and exclusion criteria were used to extract the data of eligible articles, including the first author's surname, publication year, research site, patient age, course of diabetes, sample size of each group, duration and dose of resveratrol treatment, control group medication, research methods, and postintervention data for FBG, WC, SBP, DBP, TG, and HDL-C in both groups. When the data were reported at multiple measurements, only the outcomes at the end of the intervention were included in the analysis. We also used the Finding SD provided in Cochrane Collaboration to convert and unify the data unit.

**Figure 1:**
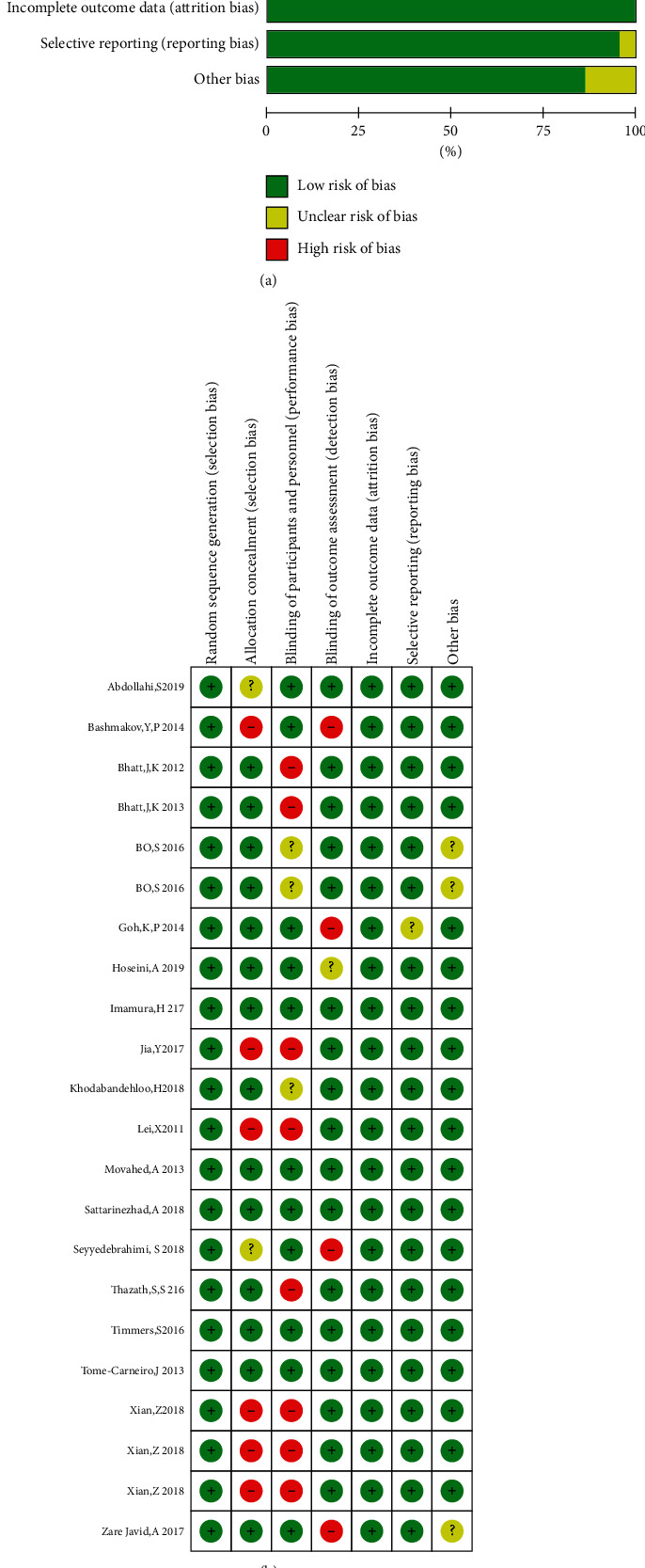
Risk of bias assessment. (a) Judgment of the bias risk for each included study. (b) Judgment of the risk of each type of bias as a percentage of all included studies.

All references cited in the eligible literature were reviewed to obtain a comprehensive collection of RCTs. According to the Cochrane methodology of systematic evaluation, we first screened each study by their title and abstract, followed by a second screening based on the full text. Disagreements were resolved by discussions with a third researcher. Literature quality was evaluated using the bias risk assessment tools provided in RevMan 5.4, including random sequence to identify selective deviation, hidden selection bias during allocation, performance bias among the participants and personnel, assessment testing bias, completeness of outcome data, biased and selective reporting, and other relevant biases. Each item was classified as “high risk” (at least one item with high bias risk), “low risk” (all items with low bias risk), and “unclear” (at least one with unclear bias risk) (Figure 1).

### 2.4. Statistical Analysis

RevMan 5.4 software was used for statistical analysis, and data were calculated as the WMD and 95% CI, with a *P*-value <0.05 indicating statistical significance. Significance across studies was defined as *I*^2^ >50% or *P* < 0.10. When *I*^2^ ≥50%, the source of heterogeneity was determined through a random-effects model if there was statistical heterogeneity among the results without clinical heterogeneity or statistical differences. Additionally, subgroup analysis was employed. We carried out subgroup analysis based on dosage of resveratrol (≤500 mg/day, 500–1000 mg/day and ≥1000 mg/day), treatment time (≤6 weeks, 6 weeks–6 months, and ≥6 months) to assess the impact of this variable on outcomes. To determine the stability of the pooled effect, one test was eliminated from the sensitivity analysis, and the summary estimate was recalculated. The WMD was used as the effect measure and calculated using the inverse variance method. It was used for units calculated using the same measurement method or obtained for the same intervention. In addition, funnel plots were used to assess publication bias. If the distribution was not symmetrical, the studies with 10 > and <20 were included. The potential publication bias was examined using Begg's rank correlation test and Egger's weighted regression test in Stata 14.0 software. *P* > 0.05 indicated a lack of publication bias.

## 3. Results

### 3.1. Literature Search Procedure

**Figure 2:**
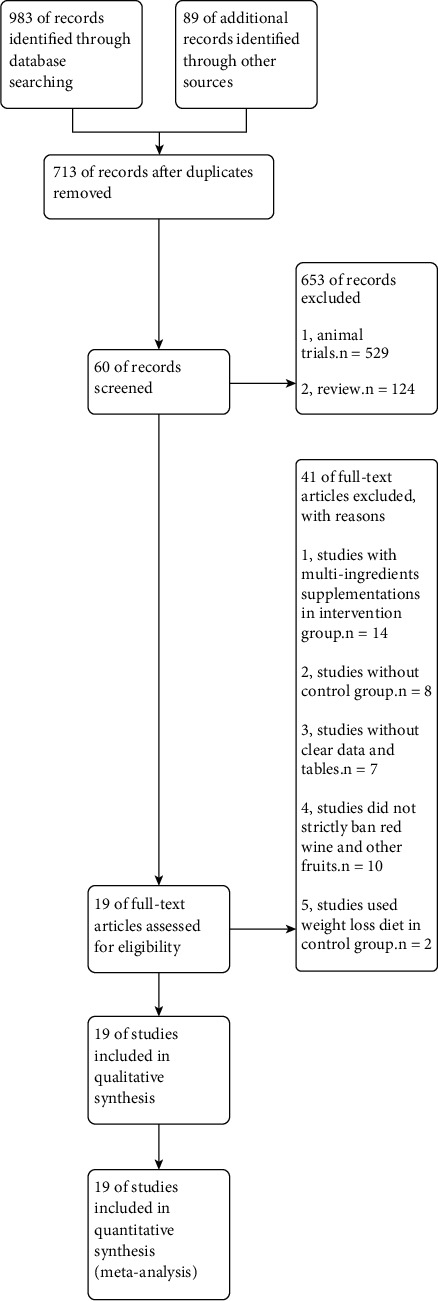
Flow chart of study selection.

A total of 1072 related documents were identified in PubMed and Scopus in addition to a manual retrieval of electronic databases and reviewing references cited in the literature. Through reading topics and abstracts, 653 animal or cell studies and reviews were excluded. Finally, 19 studies met the inclusion criteria. The studies selected by this systematic review were peer-reviewed articles and met the ethical standards, such as International Ethical Guidelines for Health-Related Research Involving Humans. The flow chart of study selection is presented in Figure 2.

### 3.2. Study Characteristics

The basic features of the studies were as follows: first author's name, year of publication, study site, patient age, course of disease, study sample size, resveratrol treatment dosage and intervention time, control group medication, and study design method. The eligible studies included seven studies conducted in Iran [19–25], three studies reported from China [26–28], two studies reported from India [29, 30], and one study each performed in Australia [31], Egypt [32], Netherlands [33], Singapore [34], Japan [35], Spain [36], and Italy [37]. The age of the patients in the studies ranged from 40.3 to 75 years, the resveratrol dosage ranged from 8.1 to 3000 mg/day, and the observation time spanned from 1.5 weeks to 12 months (Table 1).

### 3.3. Risk of Bias Assessment

The risk of bias in the 19 studies [19–37] was assessed, as presented in Figure 2. Among these studies, the risks of random sequence bias, incomplete data bias, and selective reporting bias were low. However, some studies had high risks of hidden bias in allocation and performance bias among the participants. The risk of bias was unclear for the remaining studies. Overall, the quality of the RCTs selected for this meta-analysis was acceptable.

### 3.4. Effects of Resveratrol on FBG Levels in Patients with Type 2 Diabetes Mellitus

**Figure 3:**
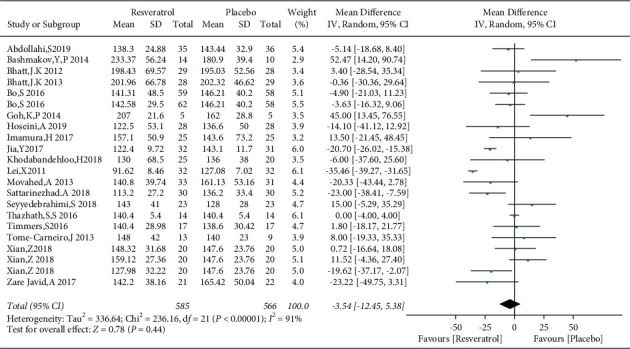
Forest plot comparing FBG levels in patients with type 2 diabetes in the control and resveratrol groups after treatment.

**Figure 4:**
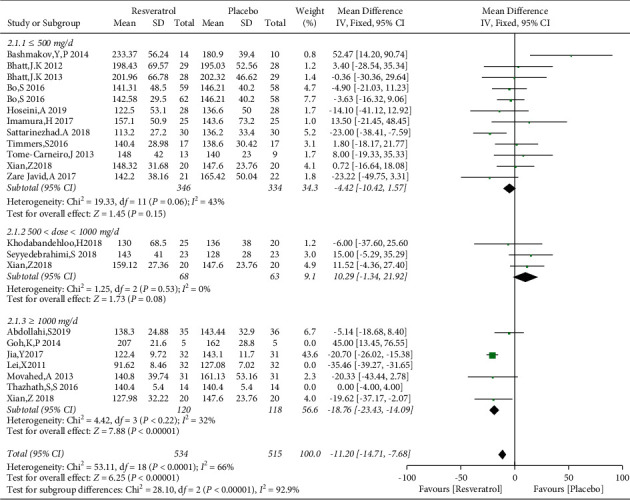
Comparison of FBG levels according to the resveratrol dose.

**Figure 5:**
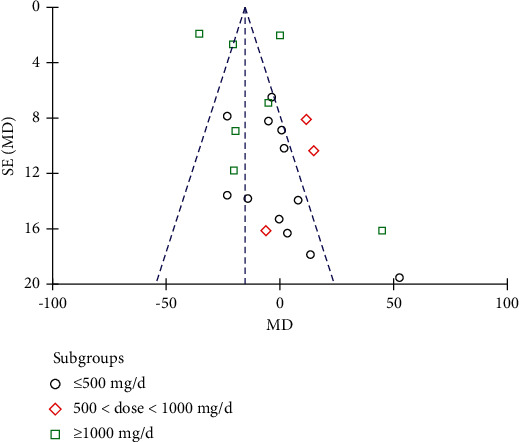
Funnel plot of the subgroup analysis of different therapeutic doses of resveratrol.

**Figure 6:**
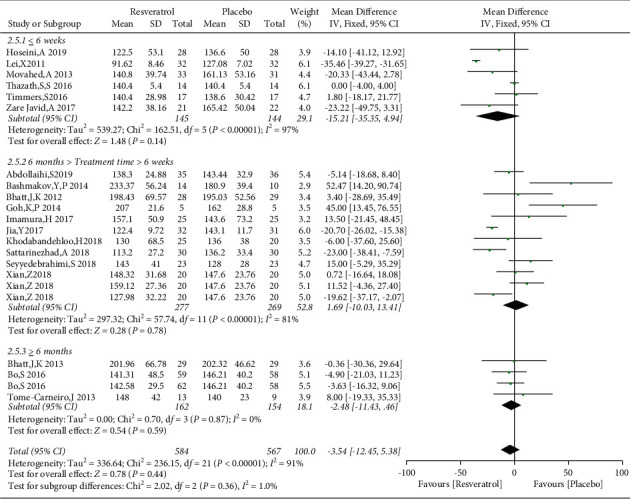
Comparison of FGB levels after treatment with resveratrol according to the duration of therapy.

**Figure 7:**
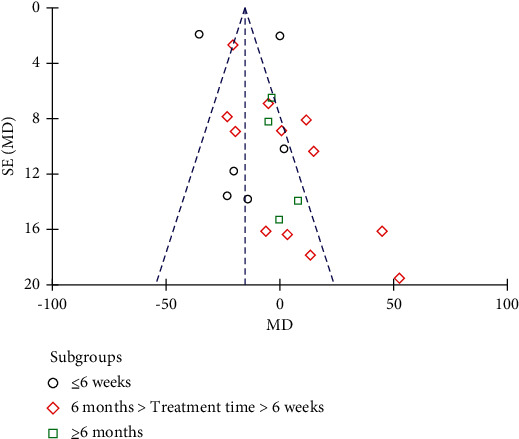
Funnel plot of the subgroup analysis of different treatment durations of resveratrol.

Nineteen studies involving 1151 subjects reported changes in the FBG levels in the control and resveratrol groups. The analysis revealed high heterogeneity (*I*^2^ = 91%). The results indicated that resveratrol did not improve FBG levels versus placebo (WMD: −3.54 mg/dL, 95% CI: −12.45, 5.38; *P*=0.44; Figure 3). We conducted a subgroup analysis of different resveratrol dosages, indicating that resveratrol had no effect on FBG levels at small (WMD: −4.42 mg/dL, 95% CI: −10.42, 1.57; *P*=0.15) and medium doses (WMD: 10.29 mg/dL, 95% CI: −1.34, 21.92; *P*=0.08). Resveratrol at doses of ≥1000 mg/day reduced FBG levels, *I*^2^ = 97%, (WMD: −18.28 mg/dL, 95% CI: −20.65, −15.91; *P* < 0.00001). However, removal of individual studies did not substantially reduce the heterogeneity, indicating that no single study was the main cause of the observed high heterogeneity. We removed two studies with an inferior quality and one study with only five participants, which reduced *I*^2^ to 32%. Therefore, these three studies were considered the main sources of heterogeneity. After removing these studies, a significant difference in favor of resveratrol was observed in the high-dose subgroup (WMD: −18.76 mg/dL, 95% CI: −23.43, −14.09; *P* < 0.00001; Figure 4). The funnel plot of the subgroup analysis of different therapeutic doses of resveratrol is presented in Figure 5. We performed a subgroup analysis of different therapeutic doses to assess the effects of resveratrol on FBG levels according to the duration of treatment. The effects of resveratrol on FBG were not associated with the duration of treatment (Figure 6). The funnel plot of the subgroup analysis of different treatment durations of resveratrol is presented in Figure 7. Egger's test revealed no publication bias (*P*=0.194).

### 3.5. The Effect of Resveratrol on WC in Patients with Type 2 Diabetes Mellitus

**Figure 8:**
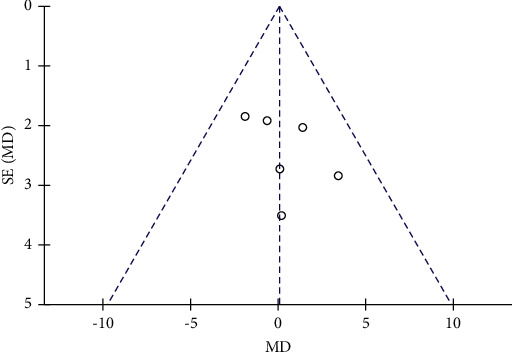
Funnel plot of changes in WC in the control and resveratrol groups.

**Figure 9:**
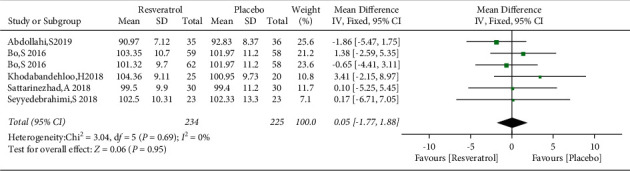
Forest plot comparing changes of WC in the control and resveratrol groups after treatment.

The changes of WC in the control and resveratrol groups were reported in five studies involving 459 subjects. The funnel plot revealed no publication bias (Figure 8) and no heterogeneity (*I*^2^ = 0%, *P*=0.69). The results showed that resveratrol had no effect on WC versus placebo (WMD: 0.05 cm, 95% CI: −1.77, 1.88; *P*=0.95; Figure 9).

### 3.6. Effects of Resveratrol on BP in Patients with Type 2 Diabetes Mellitus

**Figure 10:**
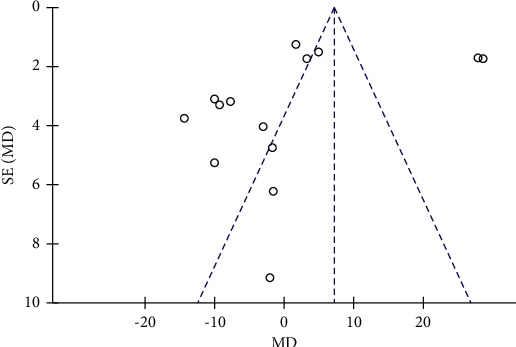
Funnel plot of changes in SBP in the control and resveratrol groups.

**Figure 11:**
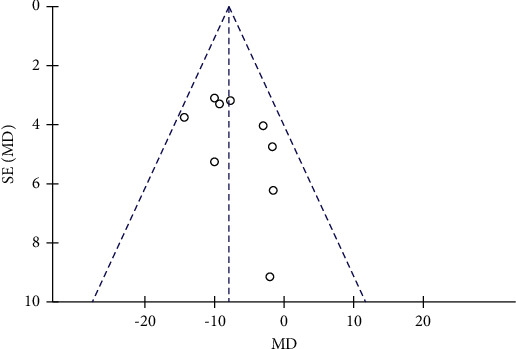
Funnel plot of SBP after removing biased literature.

**Figure 12:**
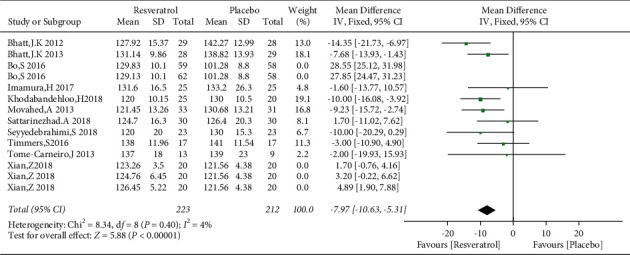
Forest map comparing SBP levels in the control and resveratrol groups after treatment.

**Figure 13:**
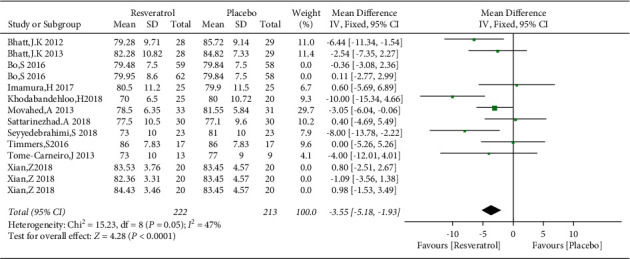
Forest plot comparing DBP levels in the control and resveratrol groups after treatment.

The changes of SBP and DBP in the control and resveratrol groups were reported in 11 studies involving 792 subjects. There was high heterogeneity in the SBP (*I*^2^ = 97%), and the funnel plots revealed publication bias (Figure 10). We used the Stata 14.0 software to conduct sensitivity analysis and observed that no single study was the major cause of heterogeneity. After removing studies by Bo et al. and Xian et al., the heterogeneity was lowered (*I*^2^ = 4%), and the funnel plots are shown in Figure 11. Resveratrol was demonstrated to improve SBP versus placebo in patients with type 2 diabetes (WMD: −7.97 mmHg, 95% CI: −10.63, −5.31; *P* < 0.00001; Figure 12). We also excluded the two studies in the assessment of DBP, resulting in low heterogeneity (*I*^2^ = 47%). The results illustrated a significant reduction in the DBP by resveratrol after removing these studies (WMD: −3.55 mmHg, 95% CI: −5.18, −1.93; *P* < 0.0001; Figure 13).

### 3.7. Effects of Resveratrol on TG and HDL-C Levels in Patients with Type 2 Diabetes Mellitus

**Figure 14:**
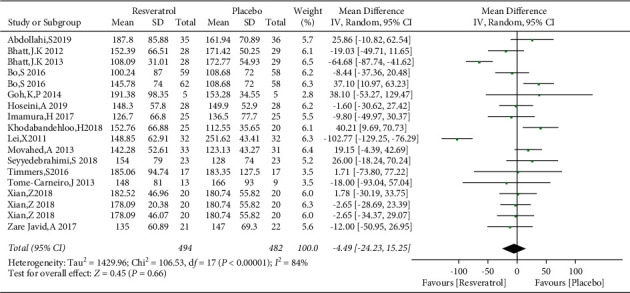
Forest plot comparing TG levels between the control and resveratrol groups after treatment.

**Figure 15:**
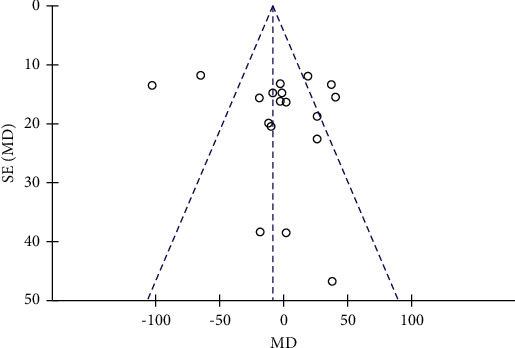
Funnel plot of changes in TG in the control and resveratrol groups.

**Figure 16:**
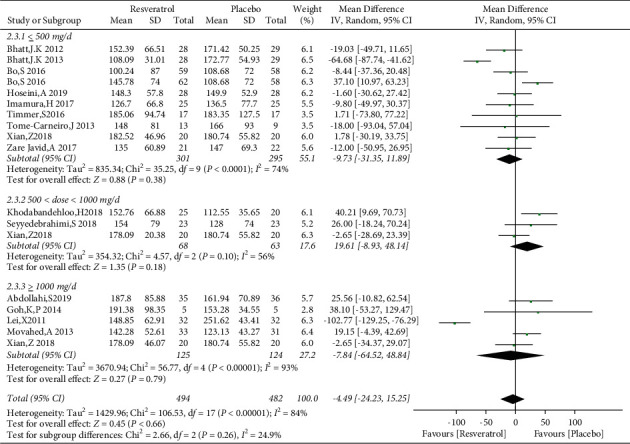
Forest plot comparing TG levels between the control and different doses of resveratrol.

**Figure 17:**
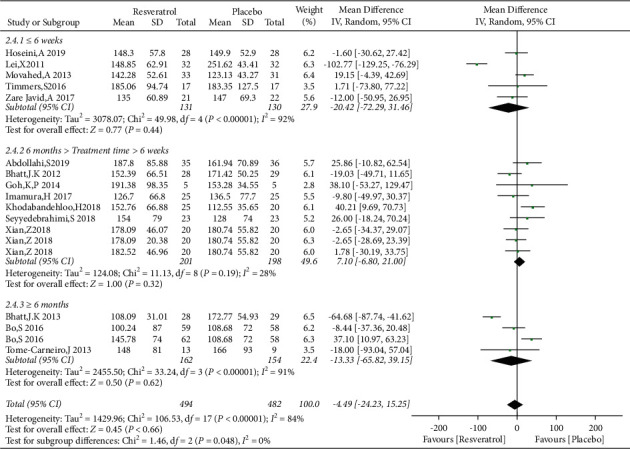
Comparison of TG levels in patients with type 2 diabetes according to the duration of resveratrol treatment.

A total of 15 studies involving 976 subjects reported changes in TG levels in the control and resveratrol groups in patients with type 2 diabetes mellitus. Because of the high heterogeneity observed (*I*^2^ = 84%), random-effects model was used. The analysis illustrated that resveratrol did not reduce TG levels versus placebo (WMD: −4.49 mg/dL, 95% CI: −24.23, 15.25; *P*=0.66; Figure 14) and the funnel plots revealed publication bias (Figure 15). In addition, subgroup analysis was conducted according to the dose and duration of resveratrol therapy. The results of subgroup analysis based on the dosage of resveratrol showed that the effect of resveratrol on TG in different dose groups was not better than that of the placebo group (Figure 16). At the same time, resveratrol was not better than the placebo group in the different treatment time groups (Figure 17). Egger's test was performed, and no publication bias was observed (*P*=0.302).

**Figure 18:**
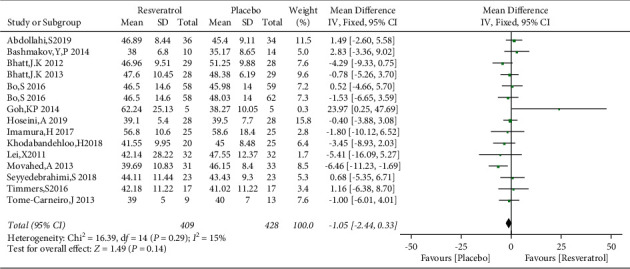
Forest plot comparing HDL-C levels between the control and resveratrol groups after treatment.

Fourteen studies involving 837 subjects reported the effects of the placebo and resveratrol on HDL levels in patients with type 2 diabetes, and low heterogeneity was identified (*I*^2^ = 15%). Compared with the placebo, resveratrol did not improve HDL-C levels (WMD: −1.05 mg/dL, 95% CI: −2.44, 0.33; *P*=0.14; Figure 18). Because of the low heterogeneity, subgroup analysis was not performed. Egger's test did not reveal a publication bias (*P*=0.364).

## 4. Discussion

Several studies on resveratrol have been published in recent years, but most of them were basic *in vitro* and *in vivo* studies of animals and cells. There are only few RCTs, and the clinical effects of the drug on relevant indicators remain unclear. Therefore, we conducted a comprehensive evaluation via systematic evaluation and meta-analysis. This study included 19 RCTs involving 1151 subjects. We observed the effects of resveratrol on metabolic parameters in patients with type 2 diabetes, including WC, FBG, SBP, DBP, TG, and HDL-C. Type 2 diabetes and metabolic syndrome often coexist; thus, it is extremely meaningful to observe these indicators in patients with type 2 diabetes. Our results illustrated that resveratrol at doses of ≥1000 mg/day resulted in better FBG control than placebo and had no effect at small and medium doses that suggest a dose-dependent effect. Meanwhile, the duration of treatment did not alter the effects of resveratrol on FBG levels. This finding slightly differs from that of Jeyaraman et al. [38] who found that resveratrol had no effect on FBG levels. These differences may be due to the inclusion of only three RCTs and the absence of subgroup analysis in their research. Tabrizi et al. [39] reported that resveratrol can reduce weight, BMI, and WC, but only in obese people. We observed that resveratrol did not improve WC in patients with type 2 diabetes. This may be due to the difference in the included population. A meta-analysis conducted by Fogacci et al. [40] reported that resveratrol had no effect on SBP and DBP, but doses >300 mg/day were beneficial in patients with cardiovascular risk factors, such as diabetes, metabolic syndrome, and obesity. This result is similar to our findings that different doses of resveratrol improved SBP and DBP in patients with type 2 diabetes. Regarding blood lipids, we concluded that resveratrol had no significant effect on TG levels in patients with type 2 diabetes. Because of the high heterogeneity, we also performed a subgroup analysis of different doses and intervention times. The results indicated that neither the dose nor the duration of resveratrol treatment affected its impact on TG levels. This is different from the conclusion of Zhao et al. [41], who reported that long-term resveratrol treatment (＞6 months) improved TG levels. This difference may be related to the inclusion of new RCTs in our study. Moreover, in the two studies included in their study with a treatment period >6 months, Bhatt et al.'s study showed that TC and TG were slightly increased after 6 months of 500 mg/day of resveratrol treatment. Moreover, only two studies involved interventions lasting at least 6 months, and more RCTs are required to reach a definitive conclusion. Our analysis also demonstrated that resveratrol was not superior to placebo concerning HDL-C control in patients with type 2 diabetes regardless of the dose or treatment duration. A new meta-analysis published by Abdelhaleem et al. [42] included data from 871 participants in 17 RCT studies showed that the application of resveratrol in patients with type 2 diabetes can improve HbA1C and SBP. When the dosage of resveratrol is ≥ 500 mg, the effect of FBG and TC is better than that of the placebo group. We think that the therapeutic dose span of 500 mg-3000 mg is relatively large, which may lead to a higher heterogeneity of the results. We conducted a subgroup analysis of 500 to 1000 mg and ≥1000 mg and found that the dose of ≥1000 mg has an effect on fasting blood glucose. However, this study was not included in the RCT study of the Chinese database, and all the meta-analyses selected random-effects models. The sensitivity analysis of heterogeneity sources and the existence of subcomponents were not detailed enough. We added a subgroup analysis of the effect of different treatment times on FBG, the effect of different treatment doses on TG, and the analysis of waist circumference indicators. Another meta-analysis by Hausenblas et al. showed that resveratrol is effective for reducing the FBG of diabetic patients. A detailed review of the meta-analysis found that only 10 RCT studies were included. We analyzed that the main reason for the difference between the results and the conclusions of our study was that those in our study did not include all RCTs [43].

Abnormal glycolipid metabolism often coexists with diabetes. With increased energy intake, excessive lipid metabolites produced following weight gain are deposited in the liver, skeletal muscle, muscle, and other tissues, resulting in decreased insulin sensitivity and increased insulin level, further promoting IR. Thus, IR is also a common pathophysiological manifestation of metabolic diseases [44]. Resveratrol can regulate SIRT1 and FOXO1, improve islet function, and reduce blood glucose levels in macaques with type 2 diabetes [45]. Basic research also revealed that resveratrol lowers blood sugar and improves IR in type 2 diabetes and protect the function of islet *β* cells by activating the insulin signaling pathway, which allows insulin to bind to insulin receptors on the cell membrane and activate insulin receptor substrate proteins [46, 47]. Resveratrol can upregulate mmu-miR-363-3p and improve IR through the PI3K-Akt pathway [48]. The study also found that resveratrol reduced blood glucose, plasma TG levels, and body weight and improved IR in high-fat diet- (HFD-) fed mice, in addition to reducing lipid accumulation, increasing glycogen storage in muscle and liver cells, and promoting lipolysis in adipocytes [49, 50]. Kaiting et al. [51] found that resveratrol administration improved the distribution of lipids, endotoxemia, inflammation, and intestinal barrier function and decreased glucose tolerance in HFD-fed mice. Resveratrol can also reduce BP in hypertensive rats by increasing sodium excretion and serum nitric oxide levels [52]. Therefore, many basic studies have confirmed the mechanisms of resveratrol in reducing blood glucose, weight, lipids, and blood pressure, but these basic studies are different from the conclusions of some RCT studies and meta-analysis mentioned earlier. These discrepancies may be related to ethnic differences, a lower number of observed cases, or dose dependency. Therefore, this study included a larger number of RCTs and greater number of patients, and its conclusions could guide future basic and clinical research.

This meta-analysis had several limitations. First, there was some heterogeneity in the data of the subjects included in this study. This heterogeneity should be analyzed in more detail to exclude the source of heterogeneity as much as possible. Second, the indicators included in the study may have bias affecting data analysis in the basic state. Third, the dose range and duration of resveratrol supplementation were large, which may have affected the conclusion. These problems will require further study.

## 5. Conclusion

This systematic review and meta-analysis provided a new piece of evidence that resveratrol treatment can reduce BP in patients with type 2 diabetes, and high-dose resveratrol can reduce FBG, although it has no effect on WC, TG, and HDL-C.

## Figures and Tables

**Table 1:** Characteristics of the included studies.

Authors (ref)	Time	Country	Sample size		Duration of diabetes (y)		Age (y)		Intervention		Study design
			Control	Intervention	Control	Intervention	Control	Intervention	Control	Intervention	
Abdollahi et al. [19]	2019	Iran	36	35	8.11 ± 6.9	9.4 ± 7.07	50.06 ± 7.69	50.14 ± 7.38	Methylcellulose	Rev 1000 mg/d 8 weeks	Double-blind, randomized, placebo-controlled, parallel trial
Bashmakov et al. [32]	2014	Egypt	10	14	15.2 ± 6.6	15.0 ± 6.9	59.8 ± 6.6	54.0 ± 10.1	Placebo	Rev 100 mg/d 60 days	Single-blind, randomized, placebo-controlled, parallel trial
Bhatt et al. [29]	2012	India	29	28	6.68 ± 4.7	7.57 ± 4.56	57.75 ± 8.71	56.67 ± 8.91	Oral hypoglycemic	Oral hypoglycemic + rev 250 mg/d 3 months	Double-blind, randomized, parallel trial
Bhatt and Nanjan [30]	2013	India	29	28	6.68 ± 4.7	7.57 ± 4.56	57.75 ± 8.71	56.67 ± 8.91	Oral hypoglycemic	Oral hypoglycemic + rev 250 mg/d 6 months	Double-blind, randomized, parallel trial
Bo et al. [37]	2016	Italy	58	59	22.6	18.5	65.4 ± 8.8	64.9 ± 8.6	Totally inert microcellulose	Rev 40 mg/d 6 months	Double-blind, randomized, placebo-controlled, parallel trial
Bo et al. [37]	2016	Italy	58	62	22.6	18.5	65.4 ± 8.8	65.0 ± 7.6	Totally inert microcellulose	Rev 500 mg/d 6 months	Double-blind, randomized, placebo-controlled, parallel trial
Goh et al. [34]	2014	Singapore	5	5	9.6 ± 6.3	9.4 ± 5.3	56.8 ± 5.3	55.8 ± 7.3	Placebo	Rev 3000 mg/d 12 weeks	Double-blind, randomized, placebo-controlled, parallel trial
Hoseini et al. [20]	2019	Iran	28	28	NA	NA	63.3 ± 10.1	61.0 ± 8.6	Placebo	Rev 500 mg/d 4 weeks	Double-blind, randomized, placebo-controlled, parallel trial
Imamura et al. [35]	2017	Japan	25	25	NA	NA	58 ± 10.1	57.4 ± 10.6	Placebo	Rev 27.97 mg/d 12 weeks	Double-blind, randomized, placebo-controlled, parallel trial
Ying et al. [26]	2017	China	31	32	1–3 y	1–3 y	64.95 ± 1.35	64.94 ± 1.36	Basic treatment	Basic treatment + rev 1000 mg/d 8 weeks	Randomized, parallel trial
Khodabandehloo et al. [21]	2018	Iran	20	25	11.25 ± 7.41	8.64 ± 6.22	61.1 ± 5.61	54.68 ± 6.72	Placebo	Rev 800 mg/d 8 weeks	Double-blind, randomized, placebo-controlled, parallel trial
Xue et al. [27]	2011	China	32	32	6.8 ± 3.4	7.7 ± 2.8	50 ± 9.7	52 ± 10.6	Basic treatment	Basic treatment + rev 1500 mg/d 4 weeks	Randomized, parallel trial
Movahed et al. [22]	2013	Iran	31	33	5.39 ± 1.36	5.81 ± 6.18	51.81 ± 6.99	52.45 ± 6.18	Totally inert microcellulose	Rev 1000 mg/d 1.5 weeks	Double-blind, randomized, placebo-controlled, parallel trial
Sattarinezhad et al. [23]	2018	Iran	30	30	**14.4** ± **6.3**	16.1 ± 6.6	55.7 ± 10.8	56.8 ± 9.7	Carboxymethylcellulose + losartan	Rev 500 mg/d + losartan 90 days	Double-blind, randomized, placebo-controlled, parallel trial
Seyyedebrahimi et al. [24]	2018	Iran	23	23	NA	NA	58.72 ± 6.06	54.96 ± 6.37	Placebo	Rev 800 mg/d 8 weeks	Double-blind, randomized, placebo-controlled, parallel trial
Thazhath et al. [31]	2016	Australia	14	14	5 ± 1	5 ± 1	67.5 ± 1.6	67.5 ± 1.6	Placebo	Rev 1000 mg/d 5 weeks	Double-blind, randomized, placebo-controlled, crossover trial
Timmers et al. [33]	2016	Netherlands	17	17	6.76 ± 3.96	6.76 ± 3.96	64 ± 7.87	64 ± 7.87	Placebo	Rev 150 mg/d 30 days	Double-blind, randomized, placebo-controlled, crossover trial
Tome-Carneiro et al. [36]	2013	Spain	9	13	NA	NA	57 ± 10	63 ± 12	Maltodextrin	Rev 8.1 mg/d 6 months 16.21 mg/d 6 months	Triple-blind, randomized, placebo-controlled, parallel trial
Zhang and Liu [28]	2018	China	20	20	NA	NA	57.6 ± 5.7	57.5 ± 5.9	Basic treatment	Basic treatment + rev 500 mg/d 12 weeks	Randomized, parallel trial
Zhang and Liu [28]	2018	China	20	20	NA	NA	57.6 ± 5.7	58.3 ± 5.7	Basic treatment	Basic treatment + rev 700 mg/d 12 weeks	Randomized, parallel trial
Zhang and Liu [28]	2018	China	20	20	NA	NA	57.6 ± 5.7	58.4 ± 4.8	Basic treatment	Basic treatment + rev 1000 mg/d 12 weeks	Randomized, parallel trial
Javid et al. [25]	2017	Iran	22	21	＜5	＜5	50.9 ± 8.9	49.1 ± 7.4	Placebo	Rev 240 mg/d 4 weeks	Double-blind, randomized, placebo-controlled, parallel trial
